# The Diagnostic Value of ECG Characteristics for Vasospastic and Microvascular Angina: A Systematic Review

**DOI:** 10.1111/anec.70003

**Published:** 2024-08-29

**Authors:** Diantha J. M. Schipaanboord, Janneke Woudstra, Yolande Appelman, Saskia Z. H. Rittersma, Tim P. van de Hoef, René van Es, Ruben Coronel, Peter Damman, Pim van der Harst, N. Charlotte Onland‐Moret, Hester M. den Ruijter

**Affiliations:** ^1^ Laboratory of Experimental Cardiology University Medical Center Utrecht, Utrecht University Utrecht The Netherlands; ^2^ Department of Cardiology, Amsterdam Cardiovascular Sciences Amsterdam UMC, Heart Centre Amsterdam The Netherlands; ^3^ Department of Cardiology, Division Heart and Lungs University Medical Center Utrecht, Utrecht University Utrecht The Netherlands; ^4^ Department of Experimental Cardiology, Amsterdam Cardiovascular Sciences University of Amsterdam, Amsterdam UMC Amsterdam The Netherlands; ^5^ Department of Cardiology Radboud University Medical Center Nijmegen The Netherlands; ^6^ Julius Center for Health Sciences and Primary Care University Medical Center Utrecht, Utrecht University Utrecht The Netherlands

**Keywords:** ANOCA, coronary microvascular dysfunction, coronary vasospasm, diagnosis, electrocardiography, sex

## Abstract

**Background:**

Coronary vascular dysfunction comprises VSA and/or MVA and is more common in women than in men with angina without obstructive coronary artery disease (ANOCA). Invasive coronary function testing is considered the reference test for diagnosis, but its burden on patients is large. We aimed to investigate the potential of electrocardiography (ECG) as noninvasive marker for vasospastic angina (VSA) and microvascular angina (MVA) diagnosis.

**Methods:**

We systematically screened Pubmed and EMBASE databases for studies reporting on ECG characteristics in ANOCA patients with (a suspicion of) coronary vascular dysfunction. We assessed study quality using QUADAS‐2. We extracted data on diagnostic values of different ECG characteristics and analyzed whether the studies were sex‐stratified.

**Results:**

Thirty publications met our criteria, 13 reported on VSA and 17 on MVA. The majority addressed repolarization‐related ECG parameters. Only 1 of the 13 VSA papers and 4 of the 17 MVA papers showed diagnostic accuracy measures of the ECG characteristics. The presence of early repolarization, T‐wave alternans, and inverted U waves showed of predictive value for VSA diagnosis. The QTc interval was predictive for MVA diagnosis in all six studies reporting on QTc interval. Sex‐stratified results were reported in only 5 of the 30 studies and 3 of those observed sex‐based differences.

**Conclusions:**

ECG features are not widely evaluated in diagnostic studies for VSA and MVA. Those features predictive for VSA and MVA diagnosis mostly point to repolarization abnormalities and may contribute to noninvasive risk stratification.

**Abbreviations**
Ach: acetylcholine
ANOCA: angina and nonobstructive coronary artery disease
CAD: coronary artery disease
CFR: coronary flow reserve
CFT: coronary function test
COVADIS: Coronary Vasomotion Disorders International Study Group
ECG: electrocardiography
EG: ergonovine
fQRS: fragmented QRS complexes
HRV: heart rate variability
MVA: microvascular angina
NLR: negative likelihood ratio
NPV: negative predictive value
PET: positron emission tomography
PLR: positive likelihood ratio
pNN50: the percentage of differences between adjacent normal RR intervals >50 ms computed during the entire 24‐h ECG
PPV: positive predictive value
SDNN: the global standard deviation of all normal RR intervals over 24‐h ECG
TRTC: total R T Cosine
TWA: T‐wave alternans
VG: ventricular gradient
VSA: vasospastic angina

## 1 Introduction

Ischemic heart disease affects 126 million people in the world (Khan et al. 2020). Obstructive coronary artery disease (CAD) is absent in 40%–70% of patients with symptoms and signs of myocardial ischemia undergoing coronary angiography (Jespersen et al. 2012). Recent studies show that the prevalence of coronary vascular dysfunction (microvascular angina [MVA] and/or vasospastic angina [VSA]) ranges between 84% and 89% among patients with persistent angina and nonobstructive CAD (ANOCA) (Ford et al. 2019; Suda et al. 2019; Konst et al. 2021). The endotype prevalence differs between sexes, with a higher prevalence of microvascular spasm and coronary microvascular dysfunction and a lower prevalence of epicardial spasm in women compared to men (Jansen et al. 2021).

The current reference standard to diagnose MVA and VSA is an invasive coronary function test (CFT). A CFT involves the administration of intracoronary acetylcholine (Ach) or ergonovine (EG) for the detection of coronary spasm. In addition, coronary flow reserve (CFR) and microvascular resistance assessment in response to adenosine using a dedicated guidewire technique is used to assess the microvascular function (Ford et al. 2018). Even though the initial presenting symptoms of these patients may be similar, the treatment and outcome depend on the endotype diagnosed (Ford et al. 2018, 2020). These findings have inspired cardiologists to perform more invasive tests which are now also recommended in clinical guidelines (Knuuti et al. 2020; Gulati et al. 2021). As a result, a broader ANOCA population is eligible to undergo invasive testing for medical treatment to be targeted to the specific underlying pathophysiological endotype. However, CFTs are of high burden for the patient and healthcare system. Unfortunately, the best patient selection strategy for CFT remains unclear, and practice standards differ across and even within countries. This highlights the need for a low‐risk non‐invasive diagnostic test for risk stratification to optimize the selection of patients in whom further testing is required.

The electrocardiogram (ECG) is an essential diagnostic tool for various cardiac pathologies, in particular myocardial ischemia. Also, ECGs are important for ischemia detection during the CFT for the diagnosis of VSA. In a nonacute setting, ECG changes were observed in cardiac diseases linked to coronary vascular dysfunction (e.g., QTc prolongation in patients with heart failure with preserved ejection fraction [Cho et al. 2021]). The ECG may therefore be a valuable noninvasive diagnostic tool for risk stratification in ANOCA patients.

We hypothesize that VSA/MVA patients compared to patients who have a noncardiac origin of their complaints present with specific ECG changes, and that these ECG changes have added value in the diagnosis of coronary vascular dysfunction in ANOCA patients. We therefore performed a systematic review presenting the current evidence for the use of ECG for the diagnosis of VSA and/or MVA in women and men with ANOCA.

## 2 Methods

### 2.1 Data Sources and Search Strategy

The search was conducted on July 7, 2021 and updated on July 29, 2022. We combined the search results from the PubMed and EMBASE databases. The search terms included words related to “electrocardiography,” “coronary microvascular dysfunction,” “coronary vasospasm,” “angina pectoris and nonobstructive coronary artery disease,” “vasomotor dysfunction” and variants on these terms (limited to title and abstract), MeSH headings, and Emtree terms. The full search strategy is listed in Method S1. We registered the protocol of this systematic review in PROSPERO (https://www.crd.york.ac.uk/prospero/; registration number: CRD42022336911).

### 2.2 Study Selection

**FIGURE 1:**
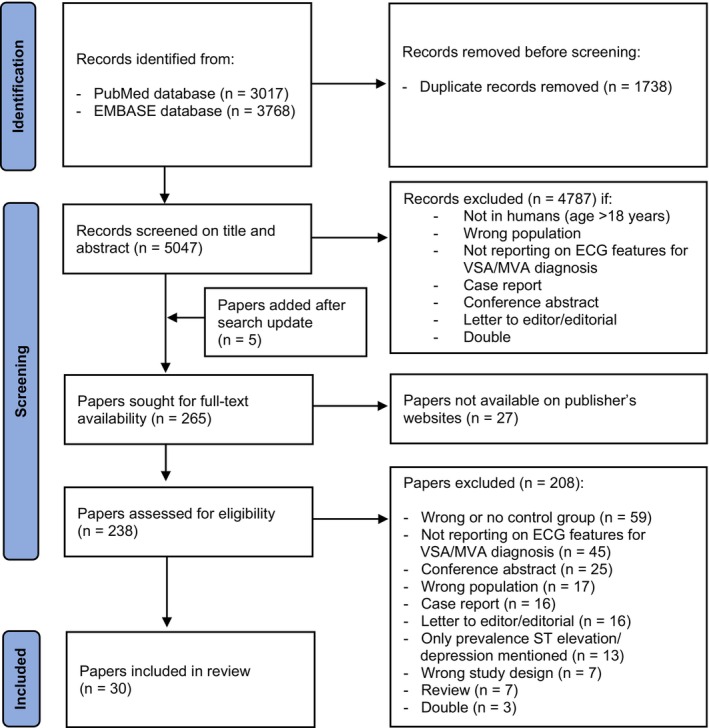
Flow diagram of the systematic search and study selection processes.

The search results were filtered to only find Dutch and English written records. After the removal of duplicates, two independent researchers (DS and JW) screened the records by title and abstract and subsequently on their full text. Only studies performed on human adults and publications for which the full text was available were considered for inclusion. All types of ECG measurements as index tests were considered eligible. Case reports, conference papers/abstracts, and editorials were excluded, as were studies that did not include patients with ANOCA, or that did not study ECG characteristics. DS and JW resolved disagreements by discussion. Figure 1 shows the search and selection processes.

### 2.3 Data Extraction

DS and JW extracted the study characteristics of interest of all included papers using a standardized data collection form. The study characteristics were the year of publication, name of the first author, country, study design, total number of participants, percentage of women in the study, average age, inclusion and exclusion criteria, description of the study group and reference group, type of ischemic heart disease, prevalence of outcome, diagnostic method/definition of the outcome, ECG measurement method, ECG features studied, and results. The definition of VSA or MVA used per study was checked against the Coronary Vasomotion Disorders International Study Group (COVADIS) criteria for a definitive diagnosis or suspicion of VSA and MVA (Beltrame et al. 2017; Ong et al. 2018). In addition, it was noted whether or not the results were stratified by sex. Reporting was done taking the PRISMA‐DTA reporting guidelines into account (McInnes et al. 2018). The data are presented separately for the two coronary vascular dysfunction endotypes (VSA and MVA). Furthermore, the level of evidence of the ECG features was rated. The level of evidence was low, moderate, or high if similar results for the ECG feature were shown in less than two cross‐sectional studies, two cross‐sectional studies, or more than two cross‐sectional studies, respectively.

### 2.4 Critical Appraisal

DS and JW independently performed the methodological quality assessment using the QUADAS‐2 tool (Whiting et al. 2011). The risk of bias was assessed on four key domains: patient selection (Domain 1), index test (Domain 2), reference standard (Domain 3), and flow and timing (Domain 4). We added the following question to Domain 2 to improve the risk of bias assessment of the index test: “Was the index test administered in the same way in all groups if a case–control design was used instead of a cross‐sectional design?” (Table S1). Publications were scored for their risk of bias (low/high/unclear) and concerns of applicability (low/medium/high/unclear). Disagreements were again resolved by discussion between DS and JW.

## 3 Results

### 3.1 Search Results

The search resulted in 6785 records, and the selection resulted in 30 papers that met the predefined inclusion and exclusion criteria as depicted in Figure 1. Of the 30 papers included in the final analysis, 13 (43%) focussed on VSA, 17 (57%) focussed on MVA, and none focussed on the combined endotype (VSA and MVA).

### 3.2 Quality Assessment

The risk of bias for the VSA papers was generally low for Domains 2 (index test), 3 (reference standard), and 4 (flow and timing) (Table 1). The risk of bias and applicability concern in Domain 1 (patient selection) were frequently high in these papers. The most common reason was the use of a case–control study design and the use of (healthy) symptom‐free controls. Furthermore, we allocated a medium or high applicability concern for Domain 2 when the aim of the study was not (exactly) the same as was aimed for in this review (i.e., the diagnostic value of an ECG characteristic for VSA or MVA). The interval between the index test (ECG) and reference standard was often not reported in both VSA and MVA papers, resulting in an unclear risk of bias (Domain 4). In addition, a high risk of bias for Domain 4 (second column) was allocated to some papers, since not all study subjects received the reference standard. Many MVA papers had a high risk of bias or applicability concern for Domain 3 (reference standard), since only a few studies used invasive coronary reactivity testing to diagnose MVA. We did not exclude studies based on their risk of bias or applicability concerns.

**TABLE 1:** Critical appraisal in accordance with the adjusted QUADAS‐2 criteria (Table S1).

First author	Year of publication	Domain 1: Patient selection				Domain 2: Index test				Domain 3: Reference standard			Domain 4: Flow and timing			
		Risk of bias			Applicability concern	Risk of bias			Applicability concern	Risk of bias		Applicability concern	Risk of bias			
VSA																
Akiya	1997	Low	Low	Low	High	Low	Low	Low	Low	Low	Low	Low	Unclear	Low	Low	Low
Igarashi	1995	High	Low	High	High	High	Low	Low	Medium	Low	Low	Low	Low	Low	Low	Low
Ikeda	2021	Low	Low	High	Low	Low	Low	Low	Low	Low	Low	Low	Low	Low	High	Low
Inamura	2015	Low	Low	Low	Unclear	Low	Low	Low	High	Low	Low	Low	Low	Low	Low	Low
Matsumoto	1989	Low	Low	Low	Low	Low	Low	Low	Low	Low	Low	Low	Low	Low	Low	Low
Miwa	1993	High	High	Low	High	Low	Low	Low	High	Low	Low	Low	Unclear	High	Low	Low
Ong	2011	Low	Low	Low	Low	Low	Low	Low	Medium	Low	Low	Low	Low	Low	Low	Low
Shimada	2012	High	High	High	High	Low	Low	Low	Medium	Low	Low	Low	Unclear	High	Low	Low
Suzuki	1998	High	High	High	High	Low	Low	Low	Medium	Low	Low	Low	Low	Low	Low	Low
Tsuchiya	1996	High	High	Low	High	Low	Low	Low	High	High	Low	High	Unclear	High	Low	Low
Yamasaki	1996	High	High	Low	High	Low	Low	Low	High	High	Low	Low	Unclear	High	Low	Low
Yano	1987	Low	Low	Low	High	Low	Low	Low	Low	Low	Low	Low	Low	Low	Low	Low
Yoshio	1993	High	High	Low	High	Low	Low	Low	High	Low	Low	Low	Unclear	High	High	Low
MVA																
Batchvarov	2002	High	High	Low	High	Low	Low	High	High	High	Low	High	Unclear	High	Low	Low
Damar	2014	High	High	Low	Low	Low	Low	Low	Low	High	Low	High	Unclear	Low	Low	Low
Dose	2018	Low	Low	Low	Low	Low	Low	Low	Low	High	Low	Low	Unclear	Low	Low	Low
Galassi	1991	High	High	Low	High	Low	Low	Low	High	High	Low	High	Unclear	High	Low	Low
Kaplan	2016	Low	Low	Low	Low	Low	Low	Low	Medium	High	Low	High	Unclear	Low	Low	Low
Lee et al. (1998)	1998	High	High	Low	High	Low	Low	Low	Low	High	Low	High	Unclear	High	Low	Low
Lee et al. (1996)	1996	High	High	Low	High	Low	Low	Low	High	High	Low	High	Unclear	High	Low	Low
Lopez	2021	Low	Low	Low	Low	Unclear	Low	Low	Low	Medium	Low	Low	High	Low	Low	Low
Mammana	1997	High	High	Low	High	Low	Low	Low	Medium	High	Low	High	Unclear	High	Low	Low
Ozcan	2021	Low	Low	Low	Low	Unclear	Low	Low	Low	Low	Low	Low	Unclear	Low	Low	Low
Rosano	1994	High	High	Low	High	Unclear	Low	Low	High	High	Low	High	Unclear	High	Low	Low
Rosen	1994	High	High	Low	High	Low	Low	Low	Medium	High	Low	High	Unclear	High	Low	Low
Roy	2020	High	High	Low	High	Low	Low	Low	High	Low	Low	Low	Unclear	High	Low	Low
Sara, Lennon et al. (2016)	2016	Low	Low	Low	Low	Low	Low	Low	Low	Low	Low	Low	Low	Low	Low	Low
Sara, Sugrue et al. (2016)	2016	Low	Low	Low	Low	Low	Low	Low	Low	Low	Low	Low	Low	Low	Low	High
Spinelli	1990	High	High	Low	High	Low	Low	Low	Medium	High	Low	High	Unclear	High	Low	Low
Youn	2005	Low	Low	Low	Low	Low	Low	Low	Low	High	Low	Low	Low	Low	Low	Low

*Note:* The green, orange, red, and gray colors represent low, medium, high, and unclear risk of bias or applicability concern, respectively.

Abbreviations: MVA, microvascular angina; VSA, vasospastic angina.

### 3.3 Vasospastic Angina

Table 2 shows an overview of the characteristics of the 13 studies describing ECGs of patients with and without VSA. Six publications used a cross‐sectional study design (*n* = 24–827 subjects, VSA prevalence between 33% and 57%), and seven used a case–control study design (*n* cases = 7–50; *n* controls = 8–50). Most studies included fewer women than men (mean: 33% women; range: 0%–58%). Only one study reported sex‐stratified results (Matsumoto et al. 1989). One article reported on the diagnostic performance of the ECG characteristic using measures of accuracy (i.e., AUC, positive predictive value [PPV], and negative predictive value [NPV], and/or sensitivity and specificity) (Matsumoto et al. 1989). The majority of the studies (*n* = 11, 85%) used an invasive spasm provocation test with Ach or EG as the reference test to diagnose VSA. In only 6 of the 13 studies, the diagnostic criteria were in accordance with the COVADIS criteria for definitive VSA diagnosis (Beltrame et al. 2017). In the 13 papers, 20 ECG features were analyzed (Table 3), including 11 heart rate variability‐related (*n* = 3 papers), two QRS‐related (*n* = 2 papers), two J‐wave‐related (*n* = 3 papers), one QT‐related (*n* = 1 paper), one T‐wave‐related (*n* = 2 papers), and three U‐wave‐related (*n* = 3 papers) parameters.

**TABLE 2:** Overview of the characteristics of the vasospastic angina studies.

First author (year) Country	Study design	*N* total (*n* VSA group)	Women (%)	Average age (years)	Prevalence of VSA (%)	Reference test	Index test	Main ECG feature(s) of study	Findings stratified by sex?
Invasive spasm provocation testing									
Yoshio et al. (1993) Japan	Case–control	18 (7)	0%	VSA: 56 RG: 50	×	Invasive spasm provocation testing by EG or Ach	24 h ambulatory two‐channel ECG recordings	Heart rate variability‐related parameters	Men only
Shimada et al. (2012) Japan	Case–control	80 (40)	55%	VSA: 59 RG: 59	×	Documentation of spontaneous episodes of chest pain, ischemic ST‐segment changes on the ECG and invasive spasm provocation testing by Ach	Ambulatory two‐channel ECG recording	T‐wave alternans	Men/women not stratified
Akiya et al. (1997) Japan	Cross‐sectional	132 (50)	18%	VSA: 56 RG (AP): 59	38%	Invasive spasm provocation testing by Ach	Vectorcardiograph signal averaged ECGs (rest, without complaints, in supine position)	Late potentials (defined as filtered QRS duration over 130 msec and/or root mean squared voltage for the last 40 msec (RMS40) below 15 uV)	Men/women not stratified
Igarashi et al. (1995) Japan	Case–control	47 (24)	26%	VSA: 57 RG: 59	×	Invasive spasm provocation testing	Precordial ECGs (lead V1 t/m V6) during provocation test	Negative U‐waves	Men/women not stratified
Inamura et al. (2015) Japan	Cross‐sectional	116 (66)	39%	VSA: 64 RG: 60	57%	Invasive spasm provocation testing by Ach	ER analysis: ECG records obtained before and during provocation test. MMA‐TWA analysis: ambulatory two‐channel (NASA and CM5) ECG recordings	T‐wave alternans and early repolarization (defined as: J point elevation > or equal to 1 mm above baseline and slurring or notching of the terminal portion of QRS at > or equal to 2 inferior and/or lateral leads)	Men/women not stratified
Ikeda et al. (2020) Japan	Cross‐sectional	286 (94)	52%	VSA: 66 RG: 63	33%	Invasive spasm provocation testing by EG or Ach	Standard 12‐lead ECG about 30 days before provocation test (without symptoms)	Early repolarization (defined as J‐point elevation > or equal to 1 mV from baseline in both or either of inferolateral leads)	Men/women not stratified
Miwa et al. (1993) Japan	Case–control	59 (21)	Unknown (VSA group: 10%)	VSA: 56 RG: unknown	×	Invasive spasm provocation testing by Ach	12‐lead ECG recorded during cold pressor test	Shape U‐wave inversion	Men/women only stratified for the VSA group
Suzuki et al. (1998) Japan	Case–control	100 (50)	43%	VSA: 58 RG: 57	×	Invasive spasm provocation testing by Ach	12‐lead ECG recorded during provocation test	QTc dispersion	Men/women not stratified
Yano et al. (1987) Japan	Cross‐sectional	102 (52)	31%	Mean unknown (range VSA: 29–70, range RG: 25–72)	×	Invasive spasm provocation testing by EG	ECG recorded during provocation test	Negative or inverted U‐waves	Men/women not stratified
Ong et al. (2011) Germany	Cross‐sectional	827 (325)	58%	VSA: 64 RG: 63	39%	Invasive spasm provocation testing by Ach.	12‐lead ECG recorded during provocation test.	Brugada ECG (type 1, 2 and 3).	Men/woen not stratified
Matsumoto et al. (1989) Japan	Cross‐sectional	24 (12)	33%	VSA: 57 RG:57	×	Invasive spasm provocation testing by EG malate	12‐lead ECG recorded during provocation test (in supine position)	QRS axis shift	Men/women stratified
Hyperventilation test and/or ST elevation									
Yamasaki et al. (1996) Japan	Case–control	18 (10)	33%	VSA: 56 RG: 58	×	Hyperventilation test	8 min lead CM5 ECG recording.	Heart rate variability‐related parameters	Men/women not stratified
Tsuchiya et al. (1996) Japan	Case–control	54 (35)	13%	VSA: 60 RG: 59	×	Resting angina associated with ST elevation more or equal to 2 mm on the ECG during spontaneous and/or hyperventilation‐induced anginal attacks	24 h two‐channel Holter ECGs	Heart rate variability‐related parameters	Men/women not stratified

Abbreviations: Ach, acetylcholine; AP, angina pectoris; CPS, chest pain syndrome; ECG, electrocardiogram; EG, ergonovine; ER, early repolarization; HF, high frequency; LF, low frequency; MMA, modified moving average; pNN50, the percentage of differences between adjacent normal RR interval >50 ms computed during the entire 24 h electrocardiogram; RG, reference group; SDNN, the global standard deviation of all normal RR intervals over 24 h; VSA, vasospastic angina.

**TABLE 3:** ECG features studied in VSA patients.

VSA	*N* sub features	Definition subfeatures	*N* papers	Main results	Quality (−/+)	Diagnostic accuracy measure(s)	Study design (*n* total)	Level of evidence
All ECG features	20		13					
Heart rate variability related	11		3					
Time domain parameters	4		2	Lower SDNN and pNN50 in VSA patients compared to controls, mean RR interval results conflicting	−	×		Low
		Mean RR interval over 4 min (Yamasaki et al. 1996)		No significant differences at three different times of day between VSA group (0.91 ± 0.04, 0.92 ± 0.04, 0.90 ± 0.04 s) and controls (0.95 ± 0.04, 0.89 ± 0.03, 0.92 ± 0.02 s)	−	×	Case–control (18)	
		Mean RR interval over 24 h (Tsuchiya et al. 1996)		Slightly lower in the VSA group compared to controls (922.1 ± 153.1 vs. 937.7 ± 112.0 ms)	−	×	Case–control (54)	
		SDNN (ms) (global standard deviation of all normal RR intervals over 24 h) (Tsuchiya et al. 1996)		Slightly but significantly lower in the VSA group compared to controls (124.2 ± 37.6 vs. 149.2 ± 29.7 ms, *p* < 0.05)	−	×	Case–control (54)	
		pNN50 (%) (percentage of differences between adjacent normal RR intervals >50 ms computed during the entire 24‐h ECG (Tsuchiya et al. 1996)		Slightly but significantly lower in the VSA group compared to controls (5.6 ± 4.6 vs. 9.4 ± 4.1%, *p* < 0.05)	−	×	Case–control (54)	
Low‐frequency domain parameters	3		3	Conflicting results	−	×		Low
		LF (0.04–0.15 Hz) normalized power of 4 min RR interval variability ([LF‐power]/[total power]‐[power of direct current component]) (Yamasaki et al. 1996)		Significantly greater in the VSA group at three different times of day (0.51 ± 0.07, 0.51 ± 0.07, 0.53 ± 0.06) than in the control group (0.25 ± 0.04, 0.31 ± 0.05, 0.31 ± 0.06)	−	×	Case–control (18)	
		LF (0.04–0.12 Hz) power of 5 min RR intervals (Yoshio et al. 1993)		No differences in LF power between VSA patients and controls	−	×	Case–control (18)	
		LF (0.04–0.15 Hz) power (ms^2^) of 24‐h RR intervals (Tsuchiya et al. 1996)		No differences in low‐frequency power between the VSA group and controls (5.6 ± 0.7 vs. 5.9 ± 0.5 ms^2^)	−	×	Case–control (54)	
High‐frequency domain parameters	3		3	No differences	−	×		Low
		HF (0.2 Hz) component coefficient of variance (CCV) of the RR‐interval variability (CCV(%) = 100*(component power)^(1/2)^/(mean RR interval)) over 4 min (Yamasaki et al. 1996)		No significant differences at three times of day between VSA groups (1.54 ± 0.36, 1.25 ± 0.19, 1.42 ± 0.29%) and controls (1.82 ± 0.48, 1.39 ± 0.30, 1.47 ± 0.36%)	−	×	Case–control (18)	
		HF (0.22–0.32 Hz) power of 5 min RR intervals (Yoshio et al. 1993)		No differences in HF power between VSA patients and controls	−	×	Case–control (18)	
		HF (0.15–0.4) Hz power (ms^2^) of 24‐h RR intervals (Tsuchiya et al. 1996)		No differences in HF power between the VSA group and controls (4.7 ± 0.9 vs. 5.1 ± 0.6 ms^2^)	−	×	Case–control (54)	
Ratio LF–HF power (LF/HF)	1		1	No differences	−	×		Low
		LF (0.04–0.15 Hz) power/HF (0.15–0.4 Hz) power over 24‐h RR interval (Tsuchiya et al. 1996)		No differences in ratio LF‐HF power between the VSA group and controls (3.1 ± 1.5 vs. 2.6 ± 0.7 ms^2^)	−	×	Case–control (54)	
QRS related	2		2					
Late potentials	1		1	Higher frequency in the VSA group compared to the non‐VSA group	+	×		**Low**
		Frequency of late potentials (%) (Akiya et al. 1997)		Higher in the VSA group (38%) than in the non‐VSA group (23%)	+	×	Cross‐sectional (132)	
(Right) QRS‐axis shift	1		1	Sensitivity of 58% and specificity of 80% right QRS axis shift for right coronary artery and left circumflex artery spasm in men and women combined	+	Men: Sensitivity = 70% (7/10) Specificity = 100% (6/6) Women: Sensitivity = 0% (0/2) Specificity = 50% (3/6)		Low
		Frequency of right QRS axis shift (Matsumoto et al. 1989)		Sensitivity of 58% and specificity of 80% right QRS axis shift for right coronary artery and left circumflex artery spasm in men and women combined	+	Men: sensitivity = 70% (7/10) specificity = 100% (6/6) Women: sensitivity = 0% (0/2) specificity = 50% (3/6)	Cross‐sectional (24)	
J‐wave related	2		3					
Early repolarization	1		2	Higher frequency of ER in VSA patients compared to non‐VSA patients	+	×		Moderate
		Frequency of ER (J‐point elevation ≥1 mm above baseline and notching or slurring of the terminal QRS portion in at least two inferior and/or lateral leads) (Ikeda et al. 2020)		Higher in VSA patients (29%) compared to non‐VSA patients (6%)	+	×	Cross‐sectional (286)	
		Frequency of ER (J‐point elevation ≥1 mm above baseline and notching or slurring of the terminal QRS portion in at least two inferior and/or lateral leads) (Inamura et al. 2015)		Higher in VSA patients (36%) compared to non‐VSA patients (12%)	+	×	Cross‐sectional (116)	
Brugada patterns	1		1	No coexistence of Brugada and VSA	+	×		Low
		Brugada patterns (Ong et al. 2011)		Prevalence of 0.3% of type 1 Brugada ECG in VSA patients	+	×	Cross‐sectional (827)	
QT related	1		1					
QTc dispersion	1		1	Greater baseline QTc dispersion in VSA patients compared to controls	−	×		Low
		Baseline QTc dispersion (Suzuki et al. 1998)		Statistically significantly greater baseline QTc dispersion before intracoronary acetylcholine injection in VSA patients compared to controls (69 ± 24 vs. 44 ± 19 ms, *p* < 0.001).	−	×	Case–control (100)	
T‐wave related	1		2					
T‐wave alternans	1		2	Higher incidences of positive TWA in the VSA group compared to the non‐VSA group or controls	−/+	×		Low
		Incidence of positive TWA (beat‐to‐beat variation >65 μV) (Shimada et al. 2012)		Higher incidence of positive TWA in the VSA group (60%) compared to controls (0%)	−	×	Case–control (80)	
		Incidence of positive TWA (beat‐to‐beat variation >65 μV) (Inamura et al. 2015)		Higher incidence of positive TWA in the VSA group (36%) compared to the non‐VSA group (12%)	+	×	Cross‐sectional (116)	
U‐wave related	3		3					
Presence of inverted U waves	2		2	Higher presence of inverted U waves in the VSA group compared to the non‐VSA group or controls	−/+	×		Low
		Presence of inverted U waves defined as new inverted U waves of ≥0.5 mm, new biphasic U waves with an initial negative of ≥0.5 mm or increased negativity of the U waves of ≥0.5 mm (Yano et al. 1987)		Higher presence of inverted U waves in the VSA group (89%) compared to the non‐VSA group (4%)	+	×	Cross‐sectional (102)	
		Presence of inverted U waves defined as negative deflection (of >0.05 mV) within the TP‐segment (Igarashi et al. 1995)		Higher presence of inverted U waves in the VSA group (79%) compared to controls (0%)	−	×	Case–control (47)	
Shape inverted U waves	1		1	Terminal shape of U‐wave inversion in the VSA group	−	×		Low
		Shape U‐wave inversion (initial or terminal) (Miwa et al. 1993)		All U‐wave inversions in the VSA group were terminal U‐wave inversions, whereas all U‐waves in the control group were initial U‐wave inversions	−	×	Case–control (59)	

Abbreviations: ECG, electrocardiography; TWA, T‐wave alternans; VSA, vasospastic angina.

#### 3.3.1 Heart Rate Variability‐Related Parameters

Heart rate variability (HRV) is considered a measure that reflects the balance between sympathetic and parasympathetic activity of the autonomic nervous system (Yamasaki et al. 1996). A low HRV can therefore indicate reduced parasympathetic activity or less commonly increased sympathetic activity. Three case–control studies (cases, *n* = 7 to 35; controls, *n* = 9 to 19; Yamasaki et al. 1996; Tsuchiya et al. 1996; Yoshio et al. 1993) focused on HRV analysis. The controls consisted of healthy subjects or subjects with atypical chest pain without any heart disease. Time‐domain parameters are based on a signal (in this case HRV) over time, whereas the frequency domain parameters describe how much of the signal (the power) lies within certain frequency bands. VSA patients and controls had no differences in high‐frequency domain HRV parameters or the ratio between low‐frequency and high‐frequency power (Table 3). The results for mean RR interval and low‐frequency domain parameters were inconsistent. In one study, the global standard deviation of all normal RR intervals over 24‐h (SDNN) and the percentage of differences between adjacent normal RR intervals >50 ms computed during the entire 24‐h ECG (pNN50) were slightly but significantly lower in VSA patients compared to controls (SDNN: 124.2 ± 37.6 ms vs. 149.2 ± 29.7 ms (*p* < 0.05), respectively; pNN50: 5.6 ± 4.6% vs. 9.4 ± 4.1% (*p* < 0.05), respectively) (Tsuchiya et al. 1996). Therefore, the evidence for a predictive value of time domain and low‐frequency domain HRV parameters for VSA is very limited (Table 3) (Yamasaki et al. 1996; Tsuchiya et al. 1996; Yoshio et al. 1993).

#### 3.3.2 QRS‐Related Parameters

Two cross‐sectional studies showed results on QRS‐related parameters, that is, late potentials and QRS axis shift (Tables 2 and 3) (Matsumoto et al. 1989; Akiya et al. 1997). The term “late potentials” is used to describe the presence of electrical activity at the end of the QRS complex. The frequency of late potentials was 38% in patients with a positive provocation test (*n* = 50) and 23% in patients with no stenosis and a negative provocation test (*n* = 82) (ns, *p* = 0.07) (Akiya et al. 1997). Right QRS axis shift showed a sensitivity and specificity of 70% and 100% for men, 0% and 50% for women, and 58% and 80% for men and women combined (*n* = 24, 33% women), respectively (Table 3) (Matsumoto et al. 1989).

#### 3.3.3 J‐Wave‐Related Parameters

Both the Brugada syndrome and the early repolarization syndrome are referred to as J‐wave syndromes and are associated with an increased risk of cardiac arrhythmias. The investigators of a cross‐sectional study (*n* subjects = 827, VSA prevalence: 39%) tested the co‐existence of Brugada syndrome and VSA (Table 2) and did not demonstrate such co‐existence (Table 3) (Ong et al. 2011). In two other cross‐sectional studies (*n* subjects = 116–286, VSA prevalence: 33%–57%), investigators explored the early repolarization syndrome in VSA and non‐VSA subjects, defined as J‐point elevation ≥1 mm above baseline and notching or slurring of the terminal QRS portion in at least two inferior and/or lateral leads. The frequency of early repolarization was higher in the VSA group (29%–36%) than in the non‐VSA group (6%–12%) (Table 3) (Ikeda et al. 2020; Inamura et al. 2015). Furthermore, the odds of VSA increased in case of the following early repolarization characteristics: early repolarization in inferior leads, notched type, and horizontal/descending ST segments and higher amplitudes (>0.2 mV) (Ikeda et al. 2020). The level of evidence for early repolarization is moderate (Table 3).

#### 3.3.4 QT‐Related Parameters

One case–control study (50 patients and 50 controls with atypical chest pain and a negative stress and spasm provocation test) focused on QTc dispersion (Table 3) (Suzuki et al. 1998). They defined QTc dispersion as the difference between the minimum and maximum QT intervals (corrected for heart rate) of a 12‐lead ECG. VSA patients had statistically significantly greater baseline QTc dispersion compared to controls (69 ± 24 ms vs. 44 ± 19 ms (*p* < 0.001), respectively) (Suzuki et al. 1998).

#### 3.3.5 T‐Wave‐Related Parameters

T‐wave alternans (TWA) is beat‐to‐beat variation in the shape and amplitude of the T wave. One cross‐sectional (Inamura et al. 2015) and one case–control (Shimada et al. 2012) study focused on TWA (Table 3). Both studies measured TWA of ambulatory ECG recordings by using the modified moving average method (Nearing and Verrier 2002). The incidence of positive TWA (value >65 μV) was higher in the VSA group (44%–60%) compared to subjects without VSA (11%) (Inamura et al. 2015) or controls that were age‐ and gender‐matched to the VSA patients (0%) (Shimada et al. 2012).

#### 3.3.6 U‐Wave‐Related Parameters

Three studies (one cross‐sectional (Yano et al. 1987) and two case–control studies (Miwa et al. 1993; Igarashi et al. 1995)) focused on the prevalence or morphology of negative or inverted U waves (Table 3). The prevalence of inverted or negative U waves was higher in the VSA group (79%–89%) compared to the non‐VSA group (4%) (Yano et al. 1987) or the control group (0%) consisting of patients with atypical chest pain without CAD or coronary spasms (Igarashi et al. 1995) (Table 3). According to one study, all U‐wave inversions in the VSA group were terminal U‐wave inversions (U‐wave inversion after positive U‐wave deflection), whereas all U‐waves were initial U‐wave inversions (U‐wave inversion proceeded to positive U‐wave deflection) in a control group of patients with hypertension without CAD (Table 3) (Miwa et al. 1993). However, the level of evidence for a predictive value of the presence of (terminal) negative and inverted U waves for VSA is low. No sex‐differentiated information was provided.

### 3.4 Microvascular Angina

Table 4 shows an overview of the characteristics of the 17 studies focussing on MVA. Of the 17 publications, five described a cross‐sectional study design (*n* = 59–926 subjects, MVA prevalence between 19% and 43%) and 12 case–control studies (*n* cases = 14–261; *n* controls = 14–261). The median percentage of women included in the studies was 64% (range 31%–100%). The percentage of women was unknown for one article. Three studies included only women. Four papers (24%) stratified their results for sex. Only five papers (29%) used invasive coronary reactivity testing with adenosine (*n* = 4 papers) or dipyridamole (*n* = 1 paper) to diagnose microvascular angina. Two (12%) used transthoracic Doppler echocardiography with dipyridamole and one (6%) a positron emission tomography (PET) scan with ^13^N‐ammonia or ^82^rubidium. In a majority of the papers (*n* = 9, 53%), the diagnosis MVA was based on a combination of symptoms and various tests (at least an exercise test and coronary angiography) to assess the presence of myocardial ischemia or rule out obstructive CAD or coronary vasospasm. In none of the studies, the diagnostic criteria were in accordance with the COVADIS criteria for definitive MVA diagnosis (Ong et al. 2018). Thirty‐eight ECG features were analyzed in the 17 publications (Table 5), including 16 heart rate variability‐related (*n* = 7 papers), three P wave‐related (*n* = 3 papers), two QRS‐related (*n* = 3 papers), two QRS‐T‐related (*n* = 1 paper), four QT‐related (*n* = 6 papers), one ST‐segment‐related (*n* = 3 papers), and ten T‐wave‐related (*n* = 3 papers) parameters (Table 5).

**TABLE 4:** Overview of the characteristics of the microvascular angina studies.

First author (year) Country	Study design	*N* total (*n* MVA group)	Women (%)	Average age (years)	Prevalence of MVA (%)	Reference test	Index test	Main ECG feature(s) of study	Findings stratified by sex?
Invasive coronary reactivity testing									
Rest/ambulatory ECG									
Roy et al. (2020) USA	Case–control	52 (36)	100%	SG: 57 RG: 51	×	Invasive coronary reactivity testing (cutoff: CFR ≤2.5)	24 h 12‐lead ambulatory ECG	ST‐segment depression (defined as more or equal to 1 min of horizontal or down‐sloping ST segment depression of more or equal to 1 mm, measured 80 ms from the J point)	Women only
Lee et al. (1996) China	Case–control	32 (18)	31%	SG: 57 RG: 54	×	Angina‐like chest pain, positive stress test, normal CAG, and no coronary spasm (12 of 18 patients had a CFR <2.5)	24‐h three dual‐channel ambulatory ECG monitoring	Heart rate variability‐related parameters	Men/women not stratified
Ozcan et al. (2021) USA	Cross‐sectional	80 (34)	69%	SG: 60 RG: 60	43%	Invasive coronary reactivity testing (cutoff: CFR <2.0)	12‐lead ECGs, ambulatory event monitors and inpatient telemetry recordings	Atrial fibrillation	Men/women not stratified
Sara, Lennon et al. 2016, Sara, Sugrue et al. (2016) USA	Cross‐sectional	926 (281)	39%	SG: 53 RG: 49	30%	Invasive coronary reactivity testing (cutoff: CFR ≤2.5)	ECGs (in rest, on the day of the reference test or within 1 week prior)	QTc prolongation	Men/women stratified
Sara, Lennon et al. 2016, Sara, Sugrue et al. (2016) USA	Case–control	522 (261)	75%	SG: 53 RG: 53	×	Invasive coronary reactivity testing (cutoff: CFR ≤2.5)	12‐lead ECG (in rest, on the day of the reference test or within 1 week prior)	T‐wave‐related parameters	Men/women stratified
PET scan									
Exercise ECG									
Lopez et al. (2021) USA	Cross‐sectional	249 (98)	64%	SG: 61 RG: 58	39%	PET scan with ^13^N‐ammonia or ^82^Rubidium as flow tracers at rest and vasodilator‐stress (cut‐off: CFR <2)	Exercise ECG	ST‐segment depression (horizontal or down‐sloping ≥1 mm)	Men/women stratified
Transthoracic echocardiography									
Exercise ECG									
Youn et al. (2005) South Korea	Cross‐sectional	59 (13)	64%	55	22%	Transthoracic Doppler echocardiography (cutoff: CFR <2.1)	12‐lead ECG (at a 3‐min interval during and after the exercise)	ST‐segment depression	Men/women not stratified
Rest ECG									
Dose (2018) Denmark	Cross‐sectional^a^	138 (26)	100%	SG: 62 RG: 62	19%	Transthoracic Doppler echocardiography (cutoff: CFR <2.0)	10‐s 12‐lead ECG (in rest, supine position)	QTc interval and T‐wave morphology parameters	Women only
Symptoms and tests									
Exercise test and/or postural changes or Valsalva maneuvres									
Spinelli et al. (1990) Italy	Case–control	34 (14)	Unknown	Mean unknown (range 45–65)	×	Symptoms, positive stress test, negative EG test, normal global and regional systolic function as assessed by echocardiography and ventriculography, and normal CAG	12‐lead ECG (in rest, at each level of exercise and at the first and 3 min of the recovery period)	Heart rate and systolic and diastolic time intervals	Men/women not stratified
Damar et al. (2014) Turkey	Case–control	84 (37)	49%	SG: 51 RG: 51	×	Symptoms, positive stress test, and normal CAG	12‐lead ECG (before exercise and at the end of each derivation)	Fragmented QRS (defined as: the presence of an additional R wave (R′), notching of the R or S wave, or the presence of fragmentation (more than one R′) in two contiguous leads corresponding to a major coronary artery)	Men/women not stratified
Mammana et al. (1997) United Kingdom	Case–control	66 (32)	100%	SG: 56 RG: 54	×	Symptoms, positive stress test, and normal CAG	Baseline 12‐lead ECG, 24‐h ambulatory ECG monitoring, and ECGs during exercise testing	QTc interval	Women only
Lee et al. (1998) Taiwan	Case–control (3 groups)	72 (26)	61%	SG: 57 RG (CAD): 57 RG (healthy): 56	×	Symptoms, positive stress test, and normal CAG	Standard 12‐lead ECGs (in different postural positions and during exercise)	QTc interval and dispersion	Men/women not stratified
Batchvarov et al. (2002) United Kingdom	Case–control	56 (16)	39%	SG: 60 RG: 33	×	Symptoms, positive stress test (ST‐segment depression), no CAD, coronary spasm or history of myocardial infarction and no valvular or myocardial disease	12‐lead digital ECGs (recorded continuously during postural changes and Valsalva maneuvers)	Ventricular gradient (VG) and Total R T cosine (TRTC)	Men/women not stratified
Rest/ambulatory ECG									
Rosano et al. (1994) United Kingdom	Case–control	46 (26)	76%	SG: 55 RG: 55	×	Symptoms, positive stress test, and normal CAG	24‐h two‐channel Holter monitoring	Heart rate variability related parameters	Men/women not stratified
Rosen et al. (1994) United Kingdom	Case–control	44 (24)	52%	SG: 55 RG: 49	×	Symptoms, positive stress test, and normal CAG	Three‐channel ECGs (in rest)	QT interval	Men/women stratified
Kaplan and Aksan (2016) Turkey	Case–control	65 (35)	42%	SG: 57 RG: 60	×	Symptoms, positive stress test and myocardial perfusion scintigraphy (MPS) with normal CAG	12‐lead ECG (in rest, supine position)	Tpeak–Tend and QT intervals	Men/women not stratified
Galassi et al. (1991) United Kingdom	Case–control (3 groups)	74 (26)	64%	SG: 51 RG (CAD): 55 RG (healthy): 47	×	Symptoms, positive stress test, normal CAG, and no evidence of coronary spasm	24‐h two‐lead ambulatory ECG monitoring (corresponding to Vs and V5)	Heart rate	Men/women not stratified

Abbreviations: CAD, coronary artery disease; CAG, coronary angiography; CFR, coronary flow reserve; CMD, coronary microvascular dysfunction; CSX, cardiac syndrome X; ECG, electrocardiogram; EG, ergonovine; MVA, microvascular angina; PET, positron emission tomography; RG, reference group; TRTC, total R T cosine; VG, ventricular gradient.

^a^ Only the data of the cross‐sectional part of the publication are used for this systematic review.

**TABLE 5:** ECG features studied in MVA patients.

MVA	*N* sub features	Definition subfeatures	*N* papers	Main results	Quality (−/+)	Diagnostic accuracy measure(s)	Study design (*n* total)	Level of evidence
All ECG features	38		17					
Heart rate variability related	16		7					
Heart rate	3		4	Higher rest heart rate in (female) MVA patients compared to (female) non‐MVA patients or controls	−/+	×		Moderate
		Heart rate (bpm) derived of 12‐lead rest ECG (Sara, Lennon et al. 2016)		Significantly higher heart rate in female MVA patients compared to female non‐MVA patients (median (Q1, Q3) bpm: 70 (60, 77) vs. 66 (58, 74), *p* = 0.019). No differences in heart rate for males with and without MVA	+	×	Cross‐sectional (926)	
		Heart rate (bpm) of 12‐lead rest ECG (Ozcan et al. 2021; Galassi et al. 1991)		Significantly higher heart rate in MVA patients compared to non‐MVA patients (78 vs. 67 bpm, *p* = 0.008)	+	×	Cross‐sectional (80)	
		Mean diurnal heart rate (bpm) derived of 24‐h two‐lead ambulatory ECG monitoring (Galassi et al. 1991)		Significantly higher mean in MVA patients compared to controls (84 ± 8 vs. 75 ± 11 bpm, *p* < 0.015).	−	×	Case–control (74)	
		Heart rate (bpm) at different exercise levels (Spinelli et al. 1990)		No significant differences between MVA patients and controls at different levels of exercise	−	×	Case–control (34)	
Heart rate/time slope	1		1	Lower in MVA patients than controls	−	×		Low
		Heart rate/time slope during exercise (Galassi et al. 1991)		Significantly lower in MVA patients compared to controls (3.3 ± 0.8 vs. 4.2 ± 1.1 bpm, *p* < 0.003)	−	×	Case–control (74)	−
Diastole percentage of the cardiac cycle	1		1	Significant shortening in MVA patients compared to controls	−	×		Low
		Diastole percentage of the cardiac cycle at different exercise levels (Spinelli et al. 1990)		Significant shortening at different exercise level in MVA patients compared to controls (±3.8%) and compared to the predicted value for the observed heart rate	−	×	Case–control (34)	−
RR interval	4		2	Lower RR interval deviations and no differences in mean RR interval between MVA patients and controls	−	×		Low
		Mean RR interval (Mammana et al. 1997)		No differences between MVA patients and controls (0.80 ± 0.13 vs. 0.84 ± 0. I7 s, *p* = ns)	−	×	Case–control (66)	
		Mean RR interval of 24‐h ECG (Rosano et al. 1994)		No differences between MVA patients and controls (787 ± 98 vs. 830 ± 83 ms, *p* = NS)	−	×	Case–control (46)	
		Standard deviations of all normal RR intervals of 24‐h ECG (Rosano et al. 1994)		Significantly lower values in MVA patients compared to controls (126 ± 22 vs. 149 ± 43 ms, *p* < 0.05)	−	×	Case–control (46)	
		Root mean square of difference of successive RR intervals (ms) (Rosano et al. 1994)		Lower values in MVA patients compared to controls (26.6 vs. 32.5 ms, *p* = ns)	−	×	Case–control (46)	
		Percentage of all adjacent normal (<50 ms in difference) RR intervals of 24‐h ECG (Rosano et al. 1994)		Significantly lower values in MVA patients compared to controls (6.3 ± 4 vs. 11.2 ± 7%, *p* < 0.05)	−	×	Case–control (46)	
Total power	2		2	Lower in MVA patients compared to controls	−	×		Low
		Total (0.01 and 1.00 Hz) power in ms^2^ (Rosano et al. 1994)		Significantly lower in MVA patients compared to controls (1273 ± 693 vs. 1790 ± 989 ms^2^, *p* < 0.05)	−	×	Case–control (46)	
		Amplitude (in ms) of total (≤0.4 Hz) spectrum (Lee et al. 1996)		Nonsignificant lower values in MVA patients compared to controls	−	×	Case–control (32)	
Low‐frequency domain parameters	3		2	Significantly lower LF power in MVA patients compared to controls	−	×		Low
		LF (0.04–0.15 Hz) power in ms^2^ (Rosano et al. 1994)		Significantly lower in MVA patients compared to controls (406 ± 176 vs. 729 ± 455 ms^2^, *p* < 0.01)	−	×	Case–control (46)	
		Amplitude (in ms) of ultralow (0.0017 to <0.0033 Hz) frequency bands (Lee et al. 1996)		Nonsignificant lower values in MVA patients compared to controls	−	×	Case–control (32)	
		Amplitude (in ms) of very low (0.0033 to <0.04 Hz) frequency bands (Lee et al. 1996)		No differences between MVA patients and controls	−	×	Case–control (32)	
		Amplitude (in ms) of low (0.04 to <0.15 Hz) frequency bands (Lee et al. 1996)		Significantly lower in MVA patients compared to controls (mean over 24 h: 406 ± 176 vs. 729 ± 455 ms^2^, *p* < 0.01)	−	×	Case–control (32)	
High‐frequency domain parameters	1		2	Lower in MVA patients compared to controls	−	×		Low
		HF (0.15–0.40 Hz) power ms^2^ (Rosano et al. 1994)		Lower in MVA patients compared to controls (155 vs. 219 ms^2^)	−	×	Case–control (46)	
		Amplitude (in ms) of high (0.15–0.40 Hz) frequency bands (Lee et al. 1996)		Significantly lower in MVA patients compared to controls between 4 and 8 PM and 12 AM–12 PM	−	×	Case–control (32)	
Ratio LF‐HF power (LF/HF)	1		2	Higher in MVA patients compared to controls	−	×		Low
		LF (0.04–0.15 Hz) power/HF (0.15–0.4 Hz) power (Rosano et al. 1994)		Higher in MVA patients compared to controls (3.6 vs. 3.4)	−	×	Case–control (46)	
		LF (0.04–0.15 Hz) power/HF (0.15–0.4 Hz) power (Lee et al. 1996)		Nonsignificant higher values in MVA patients compared to controls	−	×	Case–control (32)	
P‐wave related	3		3					
PR interval	1		3	No differences	+/−	×		High
		PR interval (Sara, Lennon et al. 2016)		No significant differences between MVA patients and non‐MVA patients (median (Q1, Q3) ms: 156 (142, 17) vs. 156 (142, 17))	+	×	Cross‐sectional (926)	
		PR interval (Dose et al. 2018)		No differences between MVA patients and non‐MVA patients (163 vs. 163 ms, *p* = ns)	−	×	Cross‐sectional (138)	
		PR interval (Ozcan et al. 2021)		No differences between MVA patients and non‐MVA patients (166 vs. 160 ms, *p* = 0.81)	+	×	Cross‐sectional (80)	
P‐wave duration	1		1	No differences	+	×		Low
		P‐wave duration (Ozcan et al. 2021)		No differences between MVA patients and non‐MVA patients (121 vs. 112 ms, *p* = 0.30)	+	×	Cross‐sectional (80)	
Atrial fibrillation	1		1	Nonsignificant higher prevalence in MVA patients compared to non‐MVA patients	+	×		Low
		Prevalence of atrial fibrillation during follow‐up (paroxysmal or nonparoxysmal AF including persistent, longstanding persistent, and permanent AF) (Ozcan et al. 2021)		Nonsignificant higher prevalence of AF in MVA patients compared to non‐MVA patients (32 vs. 15%, *p* = 0.07)	+	×	Cross‐sectional (80)	
QRS related	2		3					
Fragmented QRS	1		1	Significantly higher frequency in MVA patients compared to controls	−	×		Low
		Frequency of fragmented QRS complexes (defined as the presence of an additional R wave, notching of the R or S wave, or the presence of fragmentation (more than one R′) in two contiguous leads corresponding to a major coronary artery) (Damar et al. 2014)		Significantly higher frequency in MVA patients compared to controls (29.7% vs. 4.3%, *p* = 0.001)	−	×	Case–control (84)	
QRS duration	1		2	No differences	−/+	×		Moderate
		QRS duration (Dose et al. 2018)		No differences between MVA patients and non‐MVA patients (89 vs. 88 ms, *p* = ns)	−	×	Cross‐sectional (138)	
		QRS duration (Ozcan et al. 2021)		No differences between MVA patients and non‐MVA patients (86 vs. 86 ms, *p* = 0.28)	+	×	Cross‐sectional (80)	
QRS‐T related	2		1					
Ventricular gradient	1		1	Attenuated reaction to Valsalva maneuver and postural changes in MVA patients compared to controls	−	×		Low
		The three‐dimensional magnitude and angle of the QRS complex and T‐wave area in the ECG (Batchvarov et al. 2002)		Attenuated reaction to Valsalva maneuver and postural changes in MVA patients compared to controls	−	×	Case–control (56)	
Total R T Cosine	1		1	Attenuated reaction to Valsalva maneuver and postural changes in MVA patients compared to controls	−	×		Low
		The difference between depolarization and repolarization wavefront directions, expressed as mean cosine of the angles between QRS and T vectors in a three‐dimensional space (Batchvarov et al. 2002)		Attenuated reaction to Valsalva maneuver and postural changes in MVA patients compared to controls	−	×	Case–control (56)	
QT related	4		6					
QTc interval	2		6	Significantly higher in MVA patients compared to non‐MVA patients or controls	+/−			High
		QTc interval (time between earliest detection of depolarization in any lead to the latest detection of repolarization in any lead, corrected using Bazett's formula) (Sara, Lennon et al. 2016)		Significantly higher QTc interval in male MVA patients compared to male non‐MVA patients (median (Q1, Q3) ms: 416 (404, 428) vs. 410 (400, 421), *p* = 0.041). No significant differences in QTc interval between female MVA patients and female non‐VSA patients	+	×	Cross‐sectional (926)	
		QTc interval (beginning of the QRS complex to the end of the T‐wave corrected for heart rate using Fridericia correction (Dose et al. 2018)		Higher in MVA patients compared to non‐MVA patients (435 vs. 428 ms, *p* < 0.05)	−	×	Cross‐sectional (138)	
		QTc interval (formula for correction not given, GE Healtcare device) (Ozcan et al. 2021)		Significantly higher in MVA patients compared to non‐MVA patients (448 vs. 433 ms, *p* = 0.03)	+	×	Cross‐sectional (80)	
		Longest QT interval (onset of the QRS complex to the end of the T wave) corrected using Bazett's formula (Mammana et al. 1997)		Significantly higher QTc interval in MVA patients compared to controls (0.44 ± 0.02 vs. 0.41 ± 0.02 s, *p* = 0.0001)	−	×	Case–control (66)	
		QTc interval (onset of QRS to the end of the T wave) corrected using Bazett's formula (Lee et al. 1998)		Significantly higher maximal QTc interval in MVA patients compared to controls (456 ± 21 vs. 437 ± 31 ms, *p* = 0.04)	−	×	Case–control (72)	
		QTc interval (onset of QRS to the end of the T wave) corrected using Bazett's formula (Rosen et al. 1994)		Significantly higher in MVA patients compared to controls (439 ± 32 vs. 414 + 22 ms; *p* < 0.005). Maximal QTc tended to be longer in women than in men for both the MVA patients and controls (MVA patients: 451 ± 27 vs. 427 ± 27 ms (*p* = 0.0731); Controls: 423 ± 23 vs. 406 ± 19 ms (*p* = 0.0651))	−	×	Case–control (44)	
QTc dispersion	2		2	No differences in QTc dispersion in rest	−	QTc dispersion ≥50ms in response to standing + positive exercise test: sensitivity = 62%, specificity = 77% QTc dispersion ≥50 ms in response to postural changes: PPV = 73%, NPV = 67%		Low
		QT dispersion defined as difference between maximal and minimal QT (Rosen et al. 1994)		No difference between MVA patients and controls (38 ± 19 vs. 34 ± 9 ms, *p* = ns)	−	×	Case–control (44)	
		QTc dispersion defined as difference between maximal and minimal QTc interval (Lee et al. 1998)		No differences between MVA patients and controls (37 ± 11 vs. 36 ± 10 ms, *p* = ns). A QTc dispersion ≥50 ms in response to standing + a positive exercise test: sensitivity = 62%, specificity = 77%. QTc dispersion ≥50 ms in response to postural changes: PPV = 73%, NPV = 67%	−	QTc dispersion ≥50 ms in response to standing + positive exercise test: sensitivity = 62%, specificity = 77% QTc dispersion ≥50 ms in response to postural changes: PPV = 73%, NPV = 67%	Case–control (72)	
ST segment related	1		3					
ST depression	1		3	Higher presence in MVA patients compared to non‐MVA patients or controls	−/+	Flat or down slope ST depression in the exercise ECG: sensitivity = 23%–58%, specificity = 76%–95%		Moderate
		≥1 mm horizontal or downsloping ST segment depressions 80 ms after the J‐point in the exercise ECG (Lopez et al. 2021)		Ischemic ECG changes on the exercise ECG: sensitivity = 23% (95% CI: 15–32%), specificity = 76% (95% CI: 69–83%), PPV = 38 (95% CI:26–52) NPV = 60 (95% CI: 53–67) PLR = 0.94 (95% CI: 0.59–1.50), NLR = 1.02 (95% CI: 0.89–1.17)	+	Ischemic ECG changes on the exercise ECG: sensitivity = 23% (95% CI: 15%–32%), specificity = 76% (95% CI: 69%–83%), PPV = 38 (95% CI: 26%–52%), NPV = 60 (95% CI: 53–67), PLR = 0.94 (95% CI: 0.59–1.50), NLR = 1.02 (95% CI: 0.89–1.17)	Cross‐sectional (249)	
		Presence of flat or down slope ST depression (flat defined as ST depression of more than 1 mm, down defined as ST depression more than 1 mm 80 ms from the J‐point) in the exercise ECG (Youn et al. 2005)		Flat or down slope ST depression in the exercise ECG: sensitivity = 58%, specificity = 95%	+	Flat or down slope ST depression in the exercise ECG: sensitivity = 58%, specificity = 95%	Cross‐sectional (59)	
		At least one episodes of ST depression (≥1 min horizontal or down sloping ST segment depression ≥1.0 mm, measured 80 ms from the J point) in the ambulatory ECG (Roy et al. 2020)		Higher presence of at least one episode of ST depression in MVA patients (39% [88% asymptomatic]) compared to controls (0%)	−	×	Case–control (52)	
T‐wave related	10		3					
Tpeak‐Tend	2		2	Differences between MVA patients and controls (higher/lower in certain leads)	−/+	In males: T‐wave area in V6, T1 Y‐center of gravity in lead II and Tpeak‐Tend in lead II: sensitivity = 75%, specificity = 74%, PPV = 73%, NPV = 75%		Low
		(corrected) Tpeak‐Tend intervals (Kaplan and Aksan 2016)		Higher in MVA patients compared to controls (83.4 ± 6 vs. 75 ± 5 ms, *p* < 0.001)	−/+	×	Case–control (65)	
		Tpeak‐Tend/QT(c) ratios (Kaplan and Aksan 2016)		Higher in MVA patients compared to controls (0.20 ± 0.02 vs. 0.17 ± 0.01, *p* < 0.001)	−/+	×	Case–control (65)	
		Tpeak‐Tend interval (Sara, Sugrue et al. 2016)		Female MVA patients compared to female controls: higher in aVL and lower in V5 and V6 Male MVA patients compared to male controls: lower in II and aVF	+	In males: T‐wave area in V6, T1 Y‐center of gravity in lead II and Tpeak‐Tend in lead II: sensitivity = 75%, specificity = 74%, PPV = 73%, NPV = 75%	Case–control (522)	
T‐wave morphology parameters	8		2	T‐wave morphology differences between MVA patients and controls	−/+	In females: Right T‐wave slope in lead V6, amplitude in V6, and Y‐center of gravity in lead V1: sensitivity = 67%, specificity = 68%, PPV = 70%, NPV = 64% In males: T‐wave area in V6, T1 Y‐center of gravity in lead II, and Tpeak‐Tend in lead II: sensitivity = 75%, specificity = 74%, PPV = 73%, NPV = 75%		**Low**
		T‐wave amplitude calculated in all leads of 12‐lead ECG (Sara et al. 2016b)		Female MVA patients compared to female controls: higher in aVL and V1 Male MVA patients compared to male controls: no differences	+	In females: right T‐wave slope in lead V6, amplitude in V6, and Y‐center of gravity in lead V1: sensitivity = 67%, specificity = 68%, PPV = 70%, NPV = 64% In males: T‐wave area in V6, T1 Y‐center of gravity in lead II, and Tpeak‐Tend in lead II: sensitivity = 75%, specificity = 74%, PPV = 73%, NPV = 75%	Case–control (522)	
		T‐wave area calculated in all leads of 12‐lead ECG (Sara, Sugrue et al. 2016)		Female MVA patients compared to female controls: lower in II and V6, higher in III, aVL, and V1 Male MVA patients compared to male controls: lower in V6	+		Case–control (522)	
		T‐wave left slope calculated in all leads of 12‐lead ECG (Sara, Sugrue et al. 2016)		Female MVA patients compared to female controls: higher in V1 Male MVA patients compared to male controls: higher in II and aVF	+		Case–control (522)	
		T‐wave right slope calculated in all lead of 12‐lead ECG (Sara, Sugrue et al. 2016)		Female MVA patients compared to female controls: higher in V6, lower in V1 Male MVA patients compared to male controls: no differences	+		Case–control (522)	
		T‐wave *x*/*y*‐coordinates of the center of gravity of the T‐wave calculated in all leads of 12‐lead ECG (Sara, Sugrue et al. 2016)		Female MVA patients compared to female controls: lower X in aVF, higher Y in aVL and V1 Male MVA patients compared to male controls: no differences	+		Case–control (522)	
		T‐wave *x*/*y*‐coordinates of the center of gravity of the first 25% of the T‐wave (T1) calculated in all leads of 12‐lead ECG (Sara, Sugrue et al. 2016)		Female MVA patients compared to female controls: lower X‐coordinate in aVF, higher *Y*‐coordinate in I, III, aVR, aVL, V1–V3, and V5 Male MVA patients compared to male controls: higher *X*‐coordinate in II and aVR, higher *Y*‐coordinate in II, aVR, and aVF	+		Case–control (522)	
		T‐wave *x*/*y*‐coordinates of the center of gravity of the last 25% of the T‐wave (T4) calculated in all leads of 12‐lead ECG (Sara, Sugrue et al. 2016)		Female MVA patients compared to female controls: lower *X*‐coordinate in aVF and V5, lower *Y*‐coordinate in II, aVR, V3 and V6, higher *Y*‐coordinate in V1 Male MVA patients compared to male controls: lower *Y*‐coordinate in aVR and V6	+		Case–control (522)	
		T‐wave Morphology Combination Score (score combining three features of T‐wave morphology (flatness, asymmetry, and presence of notch), MCS = 1.6*flatness + asymmetry + notch (dimensionless) (Dose et al. 2018)		No differences between MVA patients and non‐MVA patients (73 vs. 73, *p* = ns)	−	×	Cross‐sectional (138)	

Abbreviations: ECG, electrocardiography; HF, high frequency; LF, low frequency; MVA, microvascular angina; NLR, negative likelihood ratio; NPV, negative predictive value; PLR, positive likelihood ratio; PPV, positive predictive value; VSA, vasospastic angina.

#### 3.4.1 Heart Rate Variability‐Related Parameters

Two cross‐sectional studies and five case–control studies compared heart rate (variability) features between MVA patients and controls or patients with a normal CFR (>2.0 or 2.5) (Tables 4 and 5) (Sara, Lennon et al. 2016; Spinelli et al. 1990; Galassi et al. 1991; Rosano et al. 1994; Lee et al. 1996; Ozcan et al. 2021; Mammana et al. 1997). All controls consisted of healthy subjects. One study also had a CAD control group (Galassi et al. 1991). Since the CAD group falls outside our domain, further reference will only be made to the healthy control group for this study. MVA patients had higher (diurnal) heart rates (∆ = 4–11 bpm) compared to controls or patients with normal CFR (>2.0 or 2.5) (Table 5) (Sara, Lennon et al. 2016; Galassi et al. 1991; Ozcan et al. 2021; Mammana et al. 1997). However, in one of the studies, the heart rate was only higher among female MVA patients and not among male MVA patients (Sara, Lennon et al. 2016). One of the case–control studies showed lower RR interval deviations over 24 h in MVA patients compared to controls (Rosano et al. 1994). Differences in heart rate were not seen on the ECG during exercise (Table 5) (Spinelli et al. 1990). Furthermore, MVA patients had lower diastole percentage of the cardiac cycle (±3.8%) at different levels of exercise compared to controls and shortening of the diastolic time at each exercise level compared to the predicted values based on the observed heart rate (Spinelli et al. 1990). In another study, MVA patients had a less steep heart rate/time slope during exercise than controls (3.3 ± 0.8 vs. 4.2 ± 1.1 bpm, respectively, *p* < 0.003) (Galassi et al. 1991). Regarding frequency domain HRV parameters, MVA patients had lower values of total power, low‐frequency domain and high‐frequency domain parameters and higher low‐frequency/high‐frequency power ratio compared to controls (Table 5) (Rosano et al. 1994; Lee et al. 1996). Hence, evidence for a predictive value of heart rate variability parameters for MVA is scarce.

#### 3.4.2 P‐Wave‐Related Parameters

In three cross‐sectional studies, investigators compared the PR interval (*n* = 3 papers), P‐wave duration (*n* = 1 paper), and prevalence of atrial fibrillation (*n* = 1 paper) between patients with normal and abnormal CFR (Sara, Lennon et al. 2016; Ozcan et al. 2021; Dose et al. 2018). One of these studies was designed as a case–control study but also included a cross‐sectional part. We will only describe the results from the cross‐sectional part here (Table 4) (Dose et al. 2018). None of the studies reported on sensitivity and specificity outcome measures. PR interval and P‐wave duration did not differ between patients with normal and abnormal CFR (Table 5) (Sara, Lennon et al. 2016; Ozcan et al. 2021; Dose et al. 2018). The prevalence of atrial fibrillation was 32% in patients with abnormal CFR (<2.0) and 15% in patients with normal CFR (ns, *p* = 0.07) (Table 5) (Ozcan et al. 2021).

#### 3.4.3 QRS‐Related Parameters

A case–control study showed higher frequency of fragmented QRS complexes (fQRS) in the ECGs of MVA patients (30%) compared to controls (4%, *p* = 0.001) (Table 5) (Damar et al. 2014). Two cross‐sectional studies showed no differences in QRS duration between MVA patients and patients with CFR > 2 (established with invasive coronary reactivity testing (Ozcan et al. 2021) or transthoracic Doppler echocardiography (Dose et al. 2018)) (Table 5). Some evidence exists against QRS duration and very little evidence exists for fQRS to have predictive value for MVA.

#### 3.4.4 QRS‐T‐Related Parameters

Ventricular gradient (VG) and total R T cosine (TRTC) are parameters that reflect the direction of depolarization and repolarization. The VG is the three‐dimensional magnitude and angle of the QRS complex and T‐wave area in the vector ECG. TRTC quantifies the difference between depolarization and repolarization wavefront directions, expressed as mean cosine of the angles between QRS and T vectors in a three‐dimensional space. VG and TRTC reacted attenuated to postural changes and the Valsalva maneuver (tests to examine autonomic function) in MVA patients (*n* = 16) compared to healthy subjects (*n* = 40) (Table 5) (Batchvarov et al. 2002).

#### 3.4.5 QT‐Related Parameters

QTc interval was the most studied ECG parameter of all included MVA studies (6/17 studies of which three cross‐sectional). In all six studies, MVA patients had QTc interval prolongation (mean/median ranging between 4 and 30 ms) in comparison to controls or subjects with a CFR > 2.0 or CFR > 2.5 (Table 5) (Sara, Lennon et al. 2016; Ozcan et al. 2021; Mammana et al. 1997; Dose et al. 2018; Rosen et al. 1994; Lee et al. 1998). The results were sex‐stratified in only two of the studies. In one case–control study, female MVA patients and controls had higher QTc intervals than male MVA patients and controls (Rosen et al. 1994). In a cross‐sectional study, prolongation of the QTc interval was only present among males and not among females (Sara, Lennon et al. 2016). The level of evidence for QTc interval prolongation is high.

QT(c) dispersion results are equivocal (Table 5). The QT dispersion did not differ between MVA patients and controls in one study (Rosen et al. 1994) whereas in another study, QTc dispersion ≥50 ms in response to standing plus the presence of a positive treadmill exercise test or in response to postural changes showed relatively high sensitivity (62% and 73%, respectively) and specificity (77% and 67%, respectively) for MVA diagnosis (Table 5) (Lee et al. 1998).

#### 3.4.6 ST Segment‐Related Parameters

Three studies (two cross‐sectional) focused on ST segment depression (Table 2) (Youn et al. 2005; Roy et al. 2020; Lopez et al. 2021). All defined ST segment depression as flat/horizontal or down‐sloping ST depression of more than 1 mm measured 80 ms from the J‐point (Table 5). In one of the studies, 39% of the MVA patients had at least one episode of ST depression in the ambulatory ECG compared to 0% in the reference group (88% were asymptomatic) (Roy et al. 2020). ST depression in the exercise ECG showed sensitivity values of 23% (95% CI: 15%–32%) (Lopez et al. 2021) and 58% (Youn et al. 2005), specificity values of 76% (95% CI: 69%–83%) (Lopez et al. 2021) and 95% (Youn et al. 2005), a PPV of 38 (95% CI: 26–52), an NPV of 60 (95% CI: 53–67), a positive likelihood ratio (PLR) of 0.94 (95% CI: 0.59–1.50), and a negative likelihood ratio (NLR) of 1.02 (95% CI: 0.89–1.17) (Lopez et al. 2021) for MVA diagnosis (Table 5). In one of the studies, the authors also reported the same outcome measures for women (sensitivity = 27% [95% CI: 16%–40%], specificity = 79% [95% CI: 69%–86%], PPV = 43.2 [95% CI: 27.1–60.5], NPV = 63.6 [95% CI: 54.4–72.2], PLR = 1.24 [95% CI: 0.71–2.19], NLR = 0.93 [95% CI: 0.78–1.12]). In addition, the sensitivity was lower (15%) and the specificity (87%) was higher when using stricter criteria (ST segment depression that persisted at least 1 min into recovery) (Lopez et al. 2021). The results indicate that the absence of ST segment depression in ECG can support the exclusion of MVA.

#### 3.4.7 T‐Wave‐Related Parameters

One cross‐sectional study (Dose et al. 2018) and two case–control studies (Kaplan and Aksan 2016; Sara, Sugrue et al. 2016) focused on T‐wave‐related parameters. Regarding T‐wave time aspects, Tpeak–Tend intervals differed between MVA patients and controls (Kaplan and Aksan 2016; Sara, Sugrue et al. 2016). In one study, the (corrected) Tpeak‐Tend intervals and Tpeak–Tend/QT(c) ratios were higher (Kaplan and Aksan 2016), whereas in another study, the results differed per ECG lead (Table 5) (Sara, Sugrue et al. 2016). They reported longer Tpeak–Tend intervals in aVL and shorter intervals in V5 and V6 in females. In males, they showed shorter Tpeak‐Tend intervals in lead II and aVF (Table 5) (Sara, Sugrue et al. 2016). T‐wave asymmetry, T‐wave flatness, and the presence of T‐wave notch do not seem to have predictive value for MVA, since a combined numeric score of these ECG features showed no differences between MVA (CFR < 2.0) and patients with higher CFR (Table 5) (Dose et al. 2018). Other T‐wave morphology parameters did show to be of predictive value for MVA. In females, the combination of right T slope in lead V6, T‐wave amplitude in V6 and Y‐center of gravity in V1 resulted in the highest MVA prediction accuracy (66.5%, sensitivity: 65.9%, specificity: 67.7%, PPV: 69.5%, NPV: 64%). In males, combining T‐wave area in V6, Tpeak–Tend in lead II, and T1 Y‐center of gravity in lead II predicted MVA with 74% accuracy (sensitivity: 74.6%, specificity: 73.9%, PPV: 73.4%, NPV: 75%) (Sara, Sugrue et al. 2016). Thus, T‐wave area, Y‐center of gravity (of the first 25% of the T wave), right T slope, T‐wave amplitude, and Tpeak–Tend interval may have added value in the diagnosis of MVA.

## 4 Discussion

This systematic review presents the current evidence on ECG characteristics and their predictive value for VSA or MVA in patients with ANOCA. In this review, we observed that the focus of most papers was on ventricular repolarization‐related components of the ECG (J‐waves, ST segments, and T‐ and U‐waves) and fewer on depolarization‐related ECG parameters, for both VSA and MVA. We document that ECG characteristics are not widely evaluated in diagnostic studies of both VSA (Matsumoto et al. 1989) and MVA (Lee et al. 1998; Youn et al. 2005; Lopez et al. 2021; Sara, Sugrue et al. 2016). Furthermore, only 5 (17%) of the included studies reported sex‐stratified data, and in 3 of the 5 studies sex‐based differences were discovered. Consistent findings for VSA were higher incidence of positive T‐wave alternans, higher frequency of early repolarization and inverted U waves, and the absence of differences in high‐frequency domain parameters in VSA patients compared to non‐VSA patients or controls. Consistent findings for MVA patients compared to non‐MVA or controls were higher QTc interval, lower values for total power, LF and HF domain HRV‐related parameters, and the absence of differences in PR interval and QRS duration. The results indicate that it may be possible to discriminate VSA from MVA using the ECG, since other ECG characteristics showed of predictive value for VSA (i.e., T‐wave alternans, early repolarization, and inverted U waves) than for MVA (i.e., QTc interval). The low number of studies investigating each ECG characteristic, the low sample sizes in the majority of the included studies, and the low reporting of accuracy measures make the evidence on the diagnostic performance of the ECG characteristics weak.

### 4.1 ECG Characteristics for VSA

Some evidence is available for a predictive value of T‐wave alternans, early repolarization, and inverted U waves for VSA. However, none of the studies investigating these ECG parameters reported diagnostic accuracy measures and not all of the ECG parameters were evaluated in well‐designed diagnostic studies. Differences in some time domain or low‐frequency domain HRV parameters, late potentials, right QRS axis shift, and baseline QTc dispersion are present in patients with VSA compared to non‐VSA patients or controls. The level of evidence for the predictive value of these ECG parameters was low, as these parameters were only studied in one or no cross‐sectional studies. To increase insight into the diagnostic value of these parameters, larger diagnostic test accuracy studies are necessary. High‐frequency domain HRV parameters and Brugada ECG criteria did not show promise for VSA diagnosis.

Early repolarization is thought to be caused by enhanced local early repolarization. Recently, however, it was shown that early repolarization can also be caused by local conduction delay (Boukens, Potse, and Coronel 2021). Early repolarization can therefore be caused by multiple factors, such as myocardial ischemia or structural abnormalities (Ikeda et al. 2020; Inamura et al. 2015). The higher TWA and greater baseline QTc dispersion in VSA patients compared to controls or the non‐VSA group are likely due to inhomogeneity of ventricular repolarization (Inamura et al. 2015; Suzuki et al. 1998). The higher incidence of late potentials and right QRS axis shift can be explained by slow conduction and hemiblock, respectively, caused by myocardial ischemia (Matsumoto et al. 1989; Akiya et al. 1997). Negative U waves are mentioned as (early) clinical markers of ischemia but also occur in combination with hypertension or valvular regurgitation (Yano et al. 1987; Igarashi et al. 1995). Although the mechanism behind inverted or negative U‐wave genesis is still unknown, a possible mechanism is delayed repolarization of the His‐Purkinje system (Correale et al. 2004). HRV parameters are accepted as indices of autonomic nervous activity. The high‐frequency component and low‐frequency component are thought to be indices of parasympathetic activity and sympathetic activity, respectively (Yamasaki et al. 1996). Therefore, the results of our review suggest increased sympathetic nervous activity in VSA patients (Boudou et al. 2017). The involvement of the sympathetic nervous system in the genesis of vasospasms is plausible, because this system plays an important role in the vascular tone through multiple mechanisms including direct vasoconstriction (Bruno et al. 2012).

Considering most results indicate changes in repolarization‐related ECG parameters, we recommend to focus on repolarization‐related ECG parameters for future diagnostic studies on VSA.

### 4.2 ECG Characteristics MVA

Around one third of the publications reported higher values for the QTc interval in MVA patients compared to non‐MVA patients or controls. Although these results indicate that the QTc interval has predictive value for MVA, none of these papers reported diagnostic accuracy measures. Also, higher heart rates (possibly only in females), higher presence of ST depression, and differences in Tpeak–Tend interval between MVA patients compared to non‐MVA patients and controls were observed, but only in two to three studies per ECG characteristic. PR interval and QRS duration differences were clearly absent between MVA patients and non‐MVA patients.

ECG characteristics related to myocardial ischemia or infarction, such as fQRS and ST depression, are promising to investigate as MVA patients have myocardial ischemia (Damar et al. 2014; Youn et al. 2005; Roy et al. 2020; Shehata et al. 2019). Myocardial ischemia impacts ventricular repolarization (Klabunde 2017), which can be interpreted from research by Verrier and Ikeda (2013), Verrier, Nearing, and D'Avila (2021). This can explain differences in QTc time, T‐wave duration and morphology parameters, ventricular gradient, and total R T cosine in patients with MVA. However, differences in ECG characteristics can have many other causes. For example, QTc time can also be influenced by medication, genetic or electrolyte disorders, diabetes, or a prolonged QRS duration (Rautaharju, Surawicz, and Gettes 2009). The higher heart rates, shorter diastolic times, and lower values for low‐frequency domain HRV parameters observed in patients with MVA in comparison to controls may indicate a difference in autonomic nervous activity. Since coronary flow in the subendocardial regions occurs mainly in the diastolic phase, shortening of the diastolic period can be one of the causes of regional ischemia in patients with MVA (Spinelli et al. 1990). Also, clinical syndromes associated with coronary microvascular dysfunction can contribute to ECG differences between patients with and without MVA. An example of such a clinical syndrome is heart failure with preserved ejection fraction, of which left ventricular diastolic dysfunction is considered a precursor (Camici et al. 2020). ECG features like increased QTc interval, ST segment deviation, or increased Tpeak–Tend intervals can also be observed in patients with left ventricular diastolic dysfunction (Van Ommen et al. 2021).

Indeed, most results point to changes in repolarization‐related ECG parameters in MVA patients. Therefore, studies may focus on repolarization‐related ECG parameters in diagnostic studies for MVA which seem to be the most promising based on this review.

### 4.3 Quality

The wide range of ECG features and low reporting on diagnostic performance limits the comparability of the results. Many of the included studies used a case–control study design, with controls often consisting of healthy subjects or patients without any chest pain complaints. Only two case–control studies used controls sampled from the same study base as the cases (Table 1; Kaplan and Aksan 2016; Sara, Sugrue et al. 2016). Furthermore, information on the control group was scarce in some publications. Case–control studies with wrong control groups lead to difficulties interpreting the detected associations. Using healthy control subjects will probably result in an overestimation of the associations. To be able to diagnose VSA/MVA patients in the clinical practice, VSA/MVA patients must not be compared to healthy people, but to patients who are referred for the same indication (ANOCA) but who have a noncardiac origin of their complaints.

The variation in reference standard for VSA diagnosis was low, since most studies used invasive spasm provocation testing to diagnose VSA, although using heterogeneous provocation protocols. However, the reference standard used for MVA diagnosis showed large heterogeneity, where in 12 of the 17 studies, patients were not evaluated using the diagnostic reference standard (Table 4). In 13 of the 30 studies, a proportion of patients did not even receive the reference standard used within the study (Table 1). In addition, in most studies, the diagnostic criteria for VSA or MVA were not in accordance with the COVADIS criteria, which is largely related to the fact that the COVADIS criteria had not been reported at the time of the publications (Beltrame et al. 2017; Ong et al. 2018). In seven VSA studies, the diagnostic criteria were not in accordance with the COVADIS criteria. This was due to the unreported extent of coronary artery constriction or the use of more lenient vasoconstriction thresholds of less than 90% diameter reduction to diagnose clinically significant spasms. None of the MVA studies were in accordance with the COVADIS criteria, since most studies did not specifically provide evidence of impaired coronary microvascular function and for the studies that did provide evidence of impaired coronary microvascular function, it was unclear whether the criteria of objective evidence of myocardial ischemia was met. Furthermore, the only marker of impaired coronary microvascular function in these studies was an impaired coronary flow reserve. It is therefore not known whether coronary microvascular spasm also plays a role in these patients. As a result of these considerations, it should be noted that the patients in the included papers mainly fall in the group of “suspected” of VSA or MVA according to the currently available COVADIS criteria.

The type of ECG measurement used within the included studies was also heterogeneous (Table 2). In most of the VSA papers, investigators studied ECGs measured during spasm provocation testing. Although it seems plausible that similar ECG characteristics emerge during spontaneous episodes or even that precursors are present on routinely obtained ECGs, the performance of such ECG features on ECGs needs further investigation. Our results contribute to identifying possible ECG characteristics for the diagnosis of VSA and MVA in the future. The ECG measurement methods used within the MVA studies can be roughly divided into 12‐lead rest ECG, 12‐lead ECG during exercise, and 24‐h Holter measurements. In terms of type of ECG measurement, the findings on ECG features for MVA diagnosis are therefore more generalizable for use as noninvasive diagnostic. However, the prognostic value of these ECG types will probably differ, with a higher prognostic value for exercise ECGs compared to rest ECGs (Stocco et al. 2018). Besides, in many papers, the time interval between the index test and reference standard is unclear (Table 1). This results in an unclear risk of bias. ECGs of periods before disease onset or after disease reduction due to treatment could potentially be used, but this may cause misclassification.

### 4.4 Limitations

The results are based on limited data since many included studies had a small sample size, and the studies had little overlap in studied ECG characteristics. Furthermore, only a few papers (14%) reported on the diagnostic performance of the studied ECG characteristic. Therefore, a formal meta‐analysis could not be performed. In addition, publication bias could not be assessed. Another limitation is the low number of publications (*n* = 5, 17%) reporting sex‐specific results. For most ECG features, it is therefore unclear whether they could be helpful in VSA/MVA prediction in both men and women. Furthermore, no papers reported on ECG characteristics in the combined endotype (VSA and MVA). A third of ANOCA patients undergoing CFT is affected by the combined endotype (Jansen et al. 2021). It is therefore important that this patient group is also included in research concerning diagnosis in ANOCA patients. For VSA, most publications contained data from studies in Japan, because VSA research is focused on that region. It has been described that Japanese patients have hyperreactive coronary arteries and a higher incidence of multivessel spasms in comparison to Caucasian patients (Beltrame, Sasayama, and Maseri 1999). The results may therefore not be generalizable.

### 4.5 Future Research

The included publications consist of many small case–control studies, while a cross‐sectional study design is essentially the most valid study design for diagnostic accuracy studies. Furthermore, only a small percentage of the publications (14%) reported on the diagnostic performance of the ECG characteristic by a measure of accuracy. Measures of accuracy provide us with information on the discriminative or predictive ability of a diagnostic test for a certain disease. We therefore recommend that future studies on the diagnostic performance of ECG characteristics use a cross‐sectional study design with sufficient sample size and report on diagnostic performance by measures of accuracy. In addition, we encourage the use of reporting checklists, like the STARD (STAndards for the Reporting of Diagnostic accuracy studies) Checklist (Cohen et al. 2016). The use of such a checklist can substantially improve the reporting quality of diagnostic studies and therefore improve the evidence provided. At last, we recommend the report on sex‐specific results. Of the VSA papers, only one article reported on sex‐specific results and showed that in contrast to men, the potential ECG characteristic (right QRS axis shift) did not occur in any of the women with a positive spasm provocation test. This ECG characteristic would therefore probably not be applicable for VSA diagnosis in women. This example demonstrates the importance for investigating and reporting findings in a sex‐specific manner.

## 5 Conclusion

This systematic review presents the current evidence for the use of ECG for the diagnosis of VSA or MVA in patients with ANOCA. The identification of noninvasive diagnostic ECG characteristics of VSA and MVA is important in view of the increasing population with these abnormalities. The most promising ECG features for VSA and MVA prediction are mainly related to repolarization abnormalities. To disclose latent ECG features, it can be helpful to do provocative testing during the ECG measurement, particularly in patients with MVA. However, ECG features are not widely evaluated in diagnostic studies for VSA and MVA. Due to the low number of studies investigating each ECG characteristic and the low sample sizes in the majority of the included studies, the evidence is scarce. To increase insight into the diagnostic value of electrocardiography for patients with VSA or MVA, large diagnostic studies and reporting of sex‐specific data on ECG characteristics are required.

## Author Contributions

All authors contributed to this article. D.J.M.S., J.W., Y.A., N.C.O.M., and H.M.R. were involved in the conceptualization. D.J.M.S. and J.W. conducted the literature search, screening and selection of articles, and the extraction of study characteristics. D.J.M.S. performed the analysis and wrote the first draft of the manuscript. All authors read and commented on previous versions of the manuscript. Y.A., S.Z.H.R., R.E., P.H., N.C.O.M., and H.M.R. were involved in supervision. N.C.O.M. and H.M.R. contributed equally to this work. All authors approved the final manuscript.

## Conflicts of Interest

Dr. Van de Hoef receives unrelated research funding and speaker fees from Philips, Abbott Vascular, and Boston Scientific.

## Supporting information


Data S1.

## Data Availability

The authors have nothing to report.
