# Supplementary material for: The Diagnostic Value of ECG Characteristics for Vasospastic and Microvascular Angina: A Systematic Review
Source: Ann Noninvasive Electrocardiol. 2024 Aug 29;29(5):e70003. doi: 10.1111/anec.70003 (PMC11358703; doi:10.1111/anec.70003)
Supplement: Supplementary file 1 — Data S1. [file ANEC-29-e70003-s001.pdf]

Supplemental Materials

Supplemental Method 1

Supplemental Table 1

Supplemental Method 1

Search string PubMed (3017 records), language filters: Dutch; English

("ECG"[tiab] OR "EKG"[tiab] OR "Electrocardiogr*"[tiab] OR "Elektrocardiogr*"[tiab] OR "Electro cardiogr*"[tiab] OR "Elektro cardiogr*"[tiab] OR "Body Surface Potential Map*" [tiab] OR "Body potential map*"[tiab] OR "Vectorcardiograph*"[tiab] OR “holter monitoring”[tiab] OR "Electrocardiography"[Mesh]) AND ("Microvascular angina"[tiab] OR "MVA"[tiab] OR "Coronary microvascular dysfunction"[tiab] OR "CMD"[tiab] OR "Coronary microvascular disease"[tiab] OR "MVD"[tiab] OR "X syndrome"[tiab] OR "syndrome X"[tiab] OR "CSX"[tiab] OR "coronary microcirculation"[tiab] OR "small vessel disease"[tiab] OR "Angina Pectoris with Normal Coronary Arteriogram"[tiab] OR "Microvascular Angina"[Mesh] OR "Vasospastic angina"[tiab] OR "VSA"[tiab] OR "Vasospasm*"[tiab] OR "coronary artery spasm*"[tiab] OR "coronary spasm*"[tiab] OR "microvascular spasm"[tiab] OR "Prinz-metal"[tiab] OR "Prinzmetal"[tiab] OR "Prinz metal"[tiab] OR "Variant angina"[tiab] OR "Angiospasm"[tiab] OR "Blood vessel spasm"[tiab] OR "vascular spasm"[tiab] OR "Coronary Vasospasm"[Mesh] OR "Vasomotor dysfunction"[tiab] OR "coronary dysfunction"[tiab] OR "INOCA"[tiab] OR "MINOCA"[tiab] OR "ANOCA"[tiab] OR "NOCAD"[tiab])

Search string EMBASE (3768 records), language filters: Dutch; English

('ECG':ti,ab OR 'EKG':ti,ab OR 'Electrocardiogr*':ti,ab OR 'Elektrocardiogr*':ti,ab OR 'Electro cardiogr*':ti,ab OR 'Elektro cardiogr*':ti,ab OR 'Body Surface Potential Map*':ti,ab OR 'Body potential map*':ti,ab OR 'Vectorcardiograph*':ti,ab OR 'Holter monitor*':ti,ab OR 'Electrocardiography'/exp) AND ('Microvascular angina':ti,ab OR 'MVA':ti,ab OR 'Coronary microvascular dysfunction':ti,ab OR 'CMD':ti,ab OR 'Coronary microvascular disease':ti,ab OR 'MVD':ti,ab OR 'X syndrome':ti,ab OR 'syndrome X':ti,ab OR 'CSX':ti,ab OR 'coronary microcirculation':ti,ab OR 'small vessel disease':ti,ab OR 'Angina Pectoris with Normal Coronary Arteriogram':ti,ab OR 'Syndrome X'/exp OR 'Vasospastic angina':ti,ab OR 'VSA':ti,ab OR 'Vasospasm*':ti,ab OR 'coronary artery spasm*':ti,ab OR 'coronary spasm*':ti,ab OR 'microvascular spasm':ti,ab OR 'Prinz-metal':ti,ab OR 'Prinzmetal':ti,ab OR 'Prinz metal':ti,ab OR 'Variant angina':ti,ab OR 'Angiospasm':ti,ab OR 'Blood vessel spasm':ti,ab OR 'vascular spasm':ti,ab OR 'Vasospasm'/exp OR 'Vasomotor dysfunction':ti,ab OR 'coronary dysfunction':ti,ab OR 'INOCA':ti,ab OR 'MINOCA':ti,ab OR 'ANOCA':ti,ab OR 'NOCAD':ti,ab)

Supplemental Table 1: Adjusted QUADAS-2 tool[15] used for the critical appraisal of this systematic review.

	Low	High	Medium	Unclear
**Domein 1: Patient selection**				
**A. Risk of bias**				
Was a consecutive or random sample of patients enrolled?	Same in-/exclusion criteria used for the study group and reference group.	Different in-/exclusion used for the study group and reference group.		
Was a case-control design avoided?	Cross-sectional study design or case-control study design with controls sampled from the same study base as the cases.	Case-control study design with the controls not sample from the same study base as the cases.		
Did the study avoid inappropriate exclusions?	Yes, avoided.	No, not avoided. For example if in cross-sectional studies the “difficult to diagnose” patients are excluded or in the case-control studies only patients with multivessel spasms or a very low CFR or very high IMR are taken into account.		
**B. Applicability concern**				
Is there concern that the included patients do not match the review question?	If all patients fit the domain (i.e. patients with angina complaints without obstructive coronary artery disease).	If not all patients fit the domain.		if it is unclear whether CAD is excluded in all patients.
**Domain 2: Index test**				
**A. Risk of bias**				
Were the index test results interpreted without knowledge of the results of the reference standard?	Without knowledge or with knowledge but interpreted by an algorithm.	With knowledge and interpreted by visual inspection.		If it is unclear.
If a threshold was used, was it pre-specified?	Pre-specified and not pre-specified thresholds.			
Additional question: Was the index test administered in the same way in all groups if a case-control design was used instead of a cross-sectional design?	The index test is administered in the same way for all patients.	The index test is not administered in the same way for all patients.		
**B. Applicability concern**				
Is there concern that the index test, its conduct, or interpretation differ from the review question?	The aim of the study is the same as the aim of this systematic review.	The aim of the study was different from the aim of this systematic review.	The aim of the study overlaps with the aim of this systematic review.	
**Domain 3: Reference standard**				
**A. Risk of bias**				

Is the reference standard likely to correctly classify the target condition?	Spasm provocation testing or invasive coronary reactivity testing.	Non-invasive (e.g. hyperventilation test, echo or a combination of symptoms, exclusion of CAD, ECG changes and/or stress test).		
Were the reference standard results interpreted without knowledge of the results of the index test?	Without knowledge	With knowledge.		If it is unclear.
**B. Applicability concern**				
Is there concern that the target condition as defined by the reference standard does not match the review question?	Target condition is coronary spasm or coronary microvascular dysfunction.	Target condition is variant angina or syndrome X.		
**Domain 4: Flow and timing**				
**A. Risk of bias**				
Was there an appropriate interval between index test(s) and reference standard?	Index test performed during the reference standard or within 30 days before or within 7 days after the reference standard.	Index performed >30 days before or >7 days after the reference standard.		If interval is unclear.
Did all patients receive a reference standard?	All patients received a reference standard.	Not all patients received a reference standard.		
Did patients receive the same reference standard?	All patients received the same reference standard.	Not all patients received the same reference standard. For example if in some patients ergonovine and in others acetylcholine was used for spasm provocation.		
Were all patients included in the analysis?	All patients were included in the analysis.	Not all patients were included in the analysis.		
